# Assessing responses to heat in a range-shifting, nocturnal, flying squirrel

**DOI:** 10.1093/jmammal/gyae041

**Published:** 2024-05-11

**Authors:** Vanessa R Hensley, Ek Han Tan, Emily Gagne, Danielle L Levesque

**Affiliations:** School of Biology and Ecology, University of Maine, Orono, ME 04469, United States; Ecology and Environmental Sciences, University of Maine, Orono, ME 04469, United States; School of Biology and Ecology, University of Maine, Orono, ME 04469, United States; School of Biology and Ecology, University of Maine, Orono, ME 04469, United States; Departments of Biology and Anthropology, and the Huck Institutes of the Life Sciences, Pennsylvania State University, University Park, PA 16802, United States; School of Biology and Ecology, University of Maine, Orono, ME 04469, United States

**Keywords:** critical limits, evaporative cooling, flying squirrel, Sciuridae, temperature, thermal profiles, thermal tolerance, ardilla voladora, enfriamiento evaporative, límites críticos, perfiles térmicos, Sciuridae, Temperatura, tolerancia térmica

## Abstract

Over the last few decades North American flying squirrels (*Glaucomys* spp.) have experienced dramatic northward range shifts. Previous studies have focused on the potential effects of warming winter temperatures, yet the hypothesis that rising summer temperature had a role in these range shifts remained unexplored. We therefore sought to determine the effect of high environmental temperatures on the thermoregulation and energetics of flying squirrels in an area of the Northeast of North America with a recent species turnover. Unable to find a logistically feasible population of the northern species (*Glaucomys sabrinus*), we focused on Southern Flying Squirrels (*G. volans*). Using flow-through respirometry, we measured the relationship between metabolic rate, evaporative water loss, and body temperature at high ambient temperatures. We also measured core body temperature in free-ranging flying squirrels using temperature-sensitive data loggers. We detected no significant increase in metabolic rate up to ambient temperatures as high as 40 °C. However, evaporative water loss increased at temperatures above 36.2 °C. Free-ranging body temperature of flying squirrels followed a circadian pattern with a ~2 °C difference between active and resting phase modal body temperatures. Rest-phase body temperatures were influenced by environmental temperatures with higher resting temperatures observed on days with higher daily maximum ambient temperatures but not to an extent that energy or water costs were significantly increased during rest. We found that, due to a relatively high level of thermal tolerance, high ambient temperatures are unlikely to cause an energetic strain on Southern Flying Squirrels. However, these findings do not preclude negative impacts of high ambient temperatures on the northern species, and these may still play a role in the changing distributions of *Glaucomys* in North America.

## Resumen

Las ardillas voladoras norteamericanas (*Glaucomys* sp.) han experimentado cambios dramáticos en su distribución hacia el norte durante las últimas décadas. Las investigaciones anteriores se han enfocado en los efectos potenciales del aumento de las temperaturas invernales, pero todavía no se ha explorado la hipótesis de que el aumento de la temperatura estival haya desempeñado un papel en estos cambios de rango. Por ende, deseamos determinar el efecto de las altas temperaturas ambientales sobre la termorregulación y la energía en las ardillas voladoras en una zona del noreste de América del Norte que ha mostrado un recambio de especies reciente. Estudiamos las ardillas voladoras del sur (*Glaucomys volans*), ya que no se pudo encontrar una población de la especie del norte (*Glaucomys sabrinus*) que fuera viable en términos de logística. Medimos la relación entre la tasa metabólica, la pérdida de agua por evaporación y la temperatura corporal a temperaturas ambiente altas por medio de la respirometría de flujo. Asímismo, determinamos la temperatura corporal interna de ardillas voladoras en libertad utilizando registradores de datos de temperatura. No detectamos ningún aumento estadísticamente significativo en la tasa metabólica hasta temperaturas ambiente de hasta 40 °C, pero la pérdida de agua por evaporación aumentó a temperaturas por encima de los 36,2 °C. La temperatura corporal de las ardillas voladoras en libertad siguió un patrón circadiano con una diferencia de ~2 °C entre las temperaturas corporales modales de la fase activa y de la fase de reposo. Las temperaturas ambientales afectaron las temperaturas de la fase de reposo. Los valores de dichas temperaturas fueron más altos en los días con temperaturas ambientales máximas diarias más elevadas, pero no se presentaron aumentos significativos en los costos de energía o agua durante el reposo. Determinamos que es poco probable que las altas temperaturas ambientales causen una tensión energética en las ardillas voladoras del sur debido a un nivel relativamente alto de tolerancia térmica. No obstante, estos hallazgos no excluyen los impactos negativos de las altas temperaturas ambientales sobre las especies del norte, que podrían tener un efecto sobre las distribuciones cambiantes de *Glaucomys* en América del Norte.

Rapid global changes in climate have led to observed changes to phenology, migration patterns, ranges, and life history traits in species throughout the world in tropical, temperate, and polar systems (Parmesan 2006; Pecl et al. 2017). These changes have brought to the forefront the importance of understanding the relationship between environmental temperatures, energetics, and performance in animals as species shift their ranges in response to warming temperatures (Huey et al. 2012; Urban et al. 2016). There has also been a recent push to improve bioclimatic envelope models (which rely predominantly on current abiotic conditions) by generating more physiologically informed mechanistic models (Parmesan 2006; Kearney and Porter 2009; Araújo and Peterson 2012; Briscoe et al. 2023; Conradie et al. 2023). Current associations between individual species and abiotic conditions allude to the absolute limits of physiology but do not encompass them. Physiology can therefore provide a mechanistic explanation for responses that are only hypothesized using simple bioclimatic envelope models (Seebacher and Franklin 2012; Briscoe et al. 2023).

North American flying squirrels (genus *Glaucomys*) are wide-ranging small mammals (~75 to 150 g) that occupy a variety of forest types within the temperate zone. In the past few decades (1990s to present) eastern North American Southern Flying Squirrels (*G. volans*, Linnaeus, 1758) have expanded their range northward in multiple locations, as Northern Flying Squirrels (*G. sabrinus*, Shaw, 1801) have contracted their ranges in the same direction (Bowman et al. 2005; Wood et al. 2016). The exact mechanism behind the shifts and its repercussions for forest communities are currently unknown. Researching the physiological ramifications of high temperatures on flying squirrels can better inform predictive models for both the squirrels as well as for other small mammals. Aside from acting as a potential proxy for larger species, small mammals are also important food resources for avian and mammalian carnivores and, through differential seed dispersal, can influence plant ranges (Zabel et al. 1995; Lacher et al. 2019; Mortelliti et al. 2019). Flying squirrels, specifically, offer additional advantages as a study species. They are both arboreal and nocturnal—2 characteristics that will experience climate change differently than those of ground-dwelling, diurnal species (Lovegrove et al. 2014; Bonebrake et al. 2020; Youngentob et al. 2021). As nocturnal species rest during the hottest portions of the day, they are often thought to be less vulnerable to climate change than diurnal species. However, arboreal species have more exposed nest sites and might therefore be at risk of overheating while they rest during the day (Lovegrove et al. 2014; Dausmann et al. 2023).

No definitive explanation for the observed range shifts of flying squirrels has been accepted, but several hypotheses have been proposed, all with climate change at the forefront (Weigl 1978; Pauli et al. 2004; Bowman et al. 2005; Smith 2007; Wood et al. 2016). As average global temperatures rise, both summer and winter months are getting warmer (IPCC 2021). As low winter temperatures are generally believed to define the northern limit of Southern Flying Squirrels (Muul 1974; Stapp 1992; Smith 2007), warmer winter temperatures are currently believed to be the primary driver of the recent northward range shifts of *Glaucomys* (Bowman et al. 2005; Garroway et al. 2010; Wood et al. 2016). Another proposed hypothesis suggests that changing forest composition permits the northward expansion of Southern Flying Squirrels (Lazure et al. 2016; Wood et al. 2016). As forests change from the *G. sabrinus*-preferred boreal forests to the *G. volans*-preferred temperate forests, Southern Flying Squirrels may be monopolizing on the expansion of hard mast, a primary food source. However, flying squirrel ranges are shifting much faster than those of other arboreal species, and Wood et al. (2016) found little association between forest structure and species abundance. Additionally, both species have been found outside their associated forest types and even in sympatry with one another (Weigl 1978; Smith 2007).

Warming winters might explain the northward range expansion of *G. volans* but do little to address the range contraction of *G. sabrinus*. Two additional hypotheses have emerged: direct and parasite-mediated competition. In paired nest box experiments, Southern Flying Squirrels have been observed “driving out” Northern Flying Squirrels and dominating the available tree hollows used for nest space (Weigl 1978). Northern Flying Squirrels are then forced to find nest space in areas without interspecific competition, and these areas happen to be northward.

The second hypothesis concerns the nematode parasite, *Strongyloides robustus*. Southern Flying Squirrels are known to benignly carry *S. robustus* throughout their range, but for Northern Flying Squirrels, the parasite is lethal (Wetzel and Weigl 1994; Pauli et al. 2004). If warm temperatures allow Southern Flying Squirrels to expand northward and increase their sympatry with Northern Flying Squirrels, *S. robustus* can be more easily transferred between the 2 species. Additionally, warmer temperatures encourage the persistence of *S. robustus* as it cannot reproduce in temperatures below 5 °C (Wetzel and Weigl 1994). Northern Flying Squirrels must move to areas unshared by Southern Flying Squirrels or those outside the thermal limits of *S. robustus* if they are to survive.

The aforementioned hypotheses all focus on increased winter temperatures, in one way or another, as the impetus for flying squirrel range shifts. Most previous studies have focused on thermoregulation at cold temperatures with little attention paid to the effects of high temperatures (Pearson 1947; Neumann 1967; Muul 1974; Stapp 1992; Merritt et al. 2001; Olson et al. 2017). However, as nocturnal arboreal species are known to be sensitive to increases in daytime rest-phase temperatures (Lovegrove et al. 2014; Youngentob et al. 2021) we proposed to test the potential impact of warming summer temperature on the ranges of these 2 species. High temperatures become costly as more energy is directed to cooling and taken away from other vital activities (McKechnie and Wolf 2019). This energy imbalance can prove fatal in the long run, forcing flying squirrels to adapt, move, or perish. We therefore hypothesized that rising summer temperatures may play a role in flying squirrel range shifts by increasing thermoregulatory costs during rest and that the northern species would be particularly vulnerable to high summer temperatures. To test this, we sought to characterize thermoregulatory physiology of resting flying squirrels measured under controlled laboratory conditions (using flow-through respirometry) and validate these values under field conditions using data loggers to obtain continuous values for core body temperature from free-ranging individuals.

## Materials and methods

Flying squirrels were livetrapped in the Dwight B. Demeritt Forest (44.935°N, 68.682°W) in Orono/Old Town, Maine, from May to November 2017 and May to September 2018. Based on a previous study indicating the arrival of Southern Flying Squirrels at the Holt Research Forest in Southern Maine (43.869°N, 69.776°W) in the early 1990s, the complete absence of Northern Flying Squirrels at that site since 2003 (Wood et al. 2016), as well as current range distribution maps (Cassola 2016a, 2016b), we expected to find Northern Flying Squirrels in the Demeritt Forest and Southern Flying Squirrels further south. Sherman sheet metal traps (9 × 3 × 3.5 inch Folding, Aluminum Trap, Tomahawk Live Trap, Hazelhurst, Wisconsin) were placed on the forest floor in a grid pattern of 30 to 60 traps. Grids were placed among pine–oak forest patches in both locations and individual traps were placed near large trees when possible. The traps contained cotton balls for nesting material as well as a mixture of peanut butter and rolled oats for bait. Traps were opened around dusk (1800 to 2000 h) and closed within a few hours of sunrise (0500 to 0800 h). Upon capture, flying squirrels were transferred from the trap into a handling bag to be sexed, weighed, and measured. Morphometric measurements included ear length, hindfoot length (with and without toes), forearm length, and reproductive status. Measurements were taken to the nearest millimeter using an electronic caliper and weight was recorded to the nearest gram or 0.1 g using a portable, digital scale (UNIWEIGH Digital Pocket Scale, Cochin, India). All flying squirrels were tagged using ear tags (Mouse Ear Tags, National Band and Tag Company, Newport, Kentucky) and/or passive integrated transponder tags (BioThermo13, Biomark, Boise, ID) for identification upon recapture. The transponders were inserted under the skin in the interscapular region and were used to monitor subcutaneous body temperatures during the respirometry experiments. All capture and handling procedures followed the American Society of Mammalogy guidelines for the use of wild mammals (Sikes et al. 2016) and were approved by the University of Maine’s Institutional Animal Care and Use Committee (Protocol #A2017-03-02) and the State of Maine Department of Inland Fisheries and Wildlife (Wildlife Scientific Collection Permit #2017-516).

Ambient temperature data were collected every 45 min in the North and South sections of Dwight B. Demeritt Forest from June to November 2017 and May to September 2018 in order to provide context for body temperature data and to determine the effects of ambient temperature on body temperature. Temperature-sensitive data loggers (DS1922L Thermochron iButtons, Maxim Integrated, San Jose, California) contained inside 500-mL matte black plastic bottles were placed at 3 heights in each forest—tied to a tree 3 m above the ground, affixed to the base of the tree, and buried 10 to 15 cm below the soil surface at the base of the tree. Additionally, 1 combined temperature–humidity logger (Tinytag Plus 2 TGP-4500, Gemini Data Loggers, Chichester, West Sussex, United Kingdom) was placed at ground level near the base of a tree in the Dwight B. Demeritt Forest North.

### DNA extraction and genotyping

As there is a possibility of misidentifying the species, especially in potential hybrid zones (Rogic et al. 2016), all individuals used in the respirometry experiments or with implanted data loggers (described below) had a 3-mm biopsy tissue punch (stored in 1 mL of 100% ethanol in a −20 °C freezer) taken to confirm the species via genotyping. From this, we obtained DNA for PCR using a simplified alkaline lysis method. Before extraction, the ethanol was removed and 500 µL of 50 mM NaOH and then incubated at 95 °C for 15 min. After cooling, 50 µL of 1 M Tris-HCl pH 6.8 was added, vortexed, and centrifuged before use for genotyping. To create an artificial hybrid control, 10 µL of the *G. sabrinus* was combined with 10 µL of eluted DNA from a *G. volans* sample obtained from the Holt Research Forest where the population has been confirmed to be *G. volans* since 2005 (Wood et al. 2016). Species-specific primers for *G. sabrinus*, as well as forward and reverse primers to detect cytochrome B (*Cytb*) mitochondrial gene or the cannabinoid receptor type 1 (*CNR1*) nuclear gene were prepared following Rogic et al. (2016). For each primer mix, each oligo was diluted in nuclease-free water to a final concentration of 10 μM and 1 μL of the primer mix was used per reaction. Each 15 μL PCR was prepared using GoTaq Green Mastermix (M1722, Promega) with 1 μL of DNA template. Thermal cycling was performed using the parameters as written in Rogic et al. (2016) and the products were run on a 1% TAE gel. Stained gels were imaged and used to identify the flying squirrel samples as *G. volans*, *G. sabrinus*, or a hybrid of the 2 (Supplementary Data SD1 and SD2).

### Resting metabolic rate collection and analysis

Respirometry methods are summarized briefly below with additional information provided in Supplementary Data SD3. Adult males or nonreproductive females in good condition were transported from the forest to a room on campus (<15 min) and housed for 1 to 5 h in a ventilated plastic container until placed in the respirometry chamber. All squirrels were weighed to the nearest 0.1 g before and after each experiment. Respirometry measurements (oxygen consumption, and CO_2_ and water production) were conducted during the animal’s diurnal rest phase (between 0700 and 1800 h) in a temperature-controlled cabinet set at temperatures ranging from 20 to 40 °C. Flying squirrels were exposed to a maximum of 4 temperatures during a trial. Some flying squirrels were used for multiple trials, but all trials were conducted after different capture events and at least 2 weeks apart. The first temperature upon entering the metabolic chamber was 20, 25, or 30 °C and the squirrel remained at this temperature for a minimum of 2 h. Once a squirrel was consistently resting, chamber temperature was increased by no more than 10° increments between 20 and 30 °C and 4° increments between 30 and 40 °C. Squirrels were kept at each new temperature for a minimum of 1 h. Chamber temperature was always increased, never decreased, over the course of an experiment. Experiments ranged from 4 to 8 h depending on the behavior of the squirrel and the number of temperatures tested. Squirrels were removed from the chamber if they showed signs of distress, reached a subcutaneous temperature of >41 °C, or remained active for more than 120 min. All squirrels were fed with apple and peanut butter after the completion of respirometry experiments and returned to their location of capture. In 2017, all squirrels were fasted before entering the respirometry chamber. However, fasted squirrels were less apt to rest in the metabolic chamber, and many experiments were cut short due to high locomotor activity or the persistence of stress behaviors. As our focus was on resting conditions comparable to the field and not basal conditions, in 2018 squirrels were fed a small slice of apple upon their arrival to the lab. Fed squirrels settled down more quickly inside the chamber and exhibited fewer stress behaviors throughout the experiment. The respiratory quotient between fasted and fed animals were roughly equivalent, indicating that the 2 methods were comparable.

For each 40-min stretch of measurements from the animal chamber, the lowest, continuous 5-min period of CO_2_ production was isolated using a custom macro in the analysis software (Expedata, Sable Systems, North Las Vegas, Nevada). The macro first selected the lowest 1,200 (10 min) CO_2_ values and, from that, the 600 most stable values (5 min) before calculating the mean and standard deviation of CO_2_, water vapor, and cell pressure. Some trials yielded multiple 5-min periods of low metabolic rate for a given temperature because squirrel activity and/or chamber warming time necessitated longer exposure to a given temperature. To select the best estimate of resting metabolic rate at a given temperature, each 5-min period was subjected to exclusion tests. For exclusion tests, all measurements taken within the first hour of the experiment, while the squirrel adjusted to the chamber, were excluded as were any measurements taken within an hour of a major disruption (i.e., equipment failures or adjustment, building construction, fire alarms). Additionally, periods in which the squirrel exhibited increased activity were excluded. Increased activity was detected in 1 of 2 ways: through visual confirmation using video recordings of the experiment; or when the averaged standard deviations in CO_2_ measurements across the experiment were equal to or above those of observed active periods.

Linear models were developed using the “gls” function in R Package “nlme” to evaluate the effects of various factors on metabolic rate and compared using Akaike Information Criterion (AIC_c_) scores and Akaike weights (AIC_c_Wt) generated from the “AICcmodavg” package (Mazerolle 2013; Pinheiro et al. 2013). Evaporative water loss, subcutaneous temperature, and the ratio of evaporative heat loss to metabolic heat production were all evaluated against ambient temperature. The piecewise linear regression analysis using the R Package “segmented” was applied to the best-fitting model to determine its breakpoints, indicating the lower or upper critical temperature, within a 95% confidence interval (CI; Muggeo 2008).

### Body temperature data loggers and radio-tracking

A subset of animals (>60 g, *n* = 7—all males because the females were either gestating or lactating throughout the study period) were implanted with temperature-sensitive data loggers to collect core body temperature. We used 2 types of loggers: a 1 g hermetically sealed, ceramic data logger (DST nano-T, STAR:ODDI, Gardabaer, Iceland); and a 3 to 4 g custom-made data logger (Gerhard Fluch, University of Veterinary Medicine, Vienna, Austria) coated in epoxy and a layer of surgical wax (Paramat Extra-Merck KGaA, Darmstadt, Germany). Both recorded date, time, and core body temperature every 5 min for the duration of deployment. Surgical methods are included in Supplementary Data SD3.

After completing the surgery, but while the squirrel was still sedated, we fitted the squirrels with VHF radio transmitter collars. The transmitters (~4 g, PD-2C Trasmitters, Holohil Systems Ltd, Ontario, Canada) were coated in epoxy for durability and collar wires were tucked inside rubber tubing to prevent skin irritation and were fit loose enough to rotate around the neck but not large enough to place a limb through. The squirrels were then moved to a clean container beneath a heat lamp for recovery. Additional food and analgesic were provided as they recovered from surgery. Individuals were kept in the lab overnight for observation and, after ensuring that they had both eaten and defecated, were released at the capture site the next morning. Attempts were made to recapture implanted individuals throughout the field season to monitor recovery and collect respirometry data and, if possible, locate daytime nest sites. To recover data loggers, squirrels were recaptured within 4 to 6 months and were euthanized via an Isoflurane overdose and cervical dislocation.

Body temperature (*T*_b_) was recorded every 5 min for the duration of logger deployment. Before analysis, all data were removed from the first week of deployment and any time the animal was brought to the lab for respirometry experiments. Each body temperature record was matched to an ambient temperature (*T*_a_) record and categorized as daytime or nighttime according to sunrise and sunset times in the eastern time zone using the “maptools” package in R (Bivand and Lewin-Koh, 2018). Additionally, a squirrel-defined date running from dusk to dusk was assigned to each record. The squirrel-defined date remedied the issue of splitting nocturnal activity period into 2 separate calendar days and allowed us to examine the entire activity period as 1 unit. Combining the daytime/nighttime categorization and the squirrel-defined date, the “ddply” function within the R Package “plyr” was used to calculate the mode *T*_b_ and *T*_a_, average minimum and maximum *T*_b_ and *T*_a_, and min–max *T*_b_ and *T*_a_ range for each resting and active period throughout the time series (Wickham 2011). To further characterize their body temperature patterns and to better compare with other species we also ran nonstationary waveform analysis using the “nswfa” function outlined in Levesque et al. (2017) and calculated the daily Heterothermy Index (Boyles et al. 2011). To determine factors influencing body temperature, particularly the summary statistics extracted through “plyr,” linear mixed models were developed and compared using AIC_c_ scores and AIC_c_Wt (Supplementary Data SD3). All statistics were performed using R version 3.3 or 4.2.2 (R Development Core Team 2018) and model assumptions were verified by observing qq plots and histograms of the residuals.

## Results

**Fig. 1.**
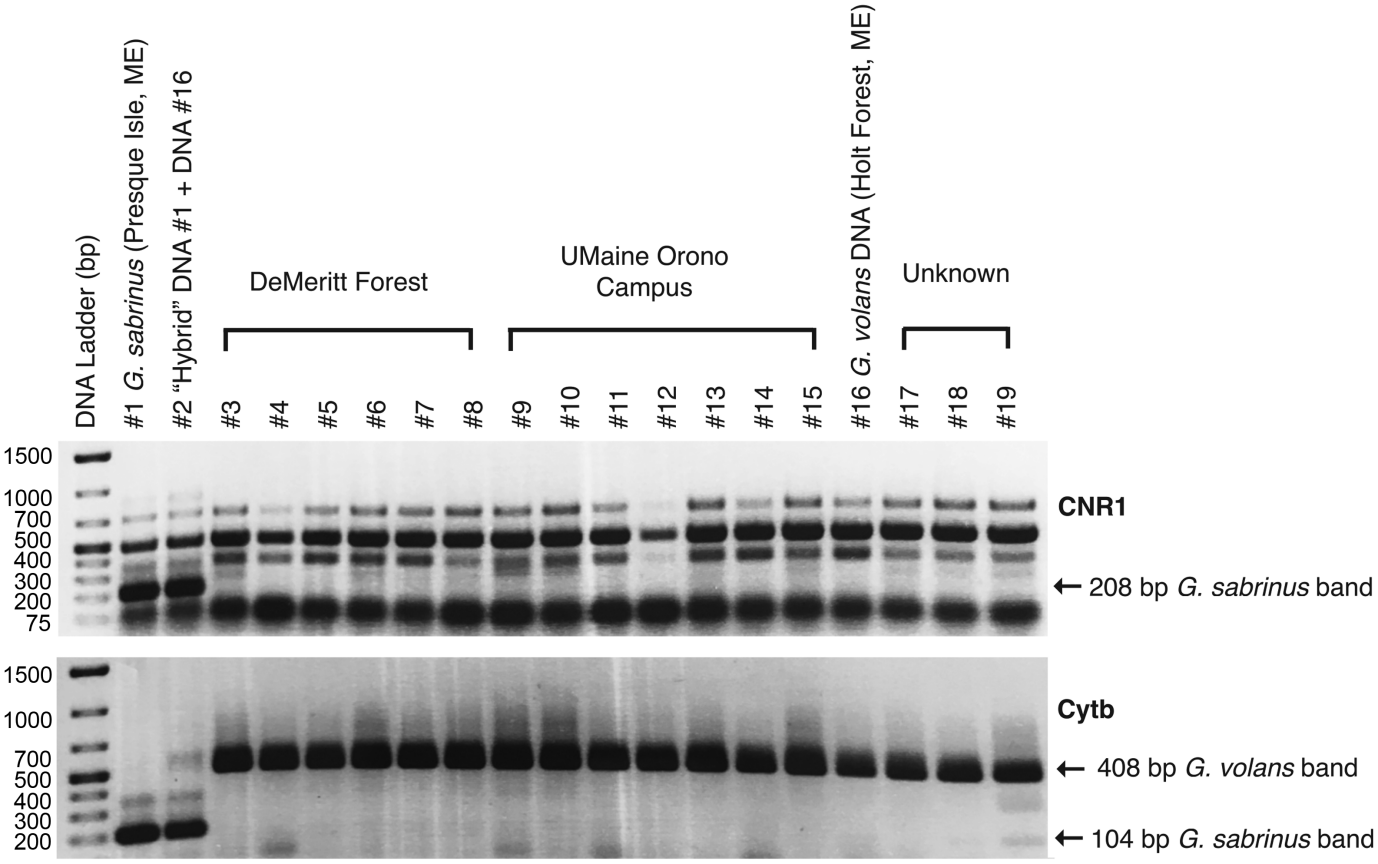
Multiplex PCR gel for *CNR1* and *Cytb* for flying squirrel samples from this study. Sample #1 is the *Glaucomys sabrinus* positive control from northern Maine and Sample #16 is the *G. volans* positive control from southern Maine. Sample #2 is a “hybrid” sample containing equivolumes of DNA from Sample #1 and #16. For *CNR1*, a 208-bp band specific to *G. sabrinus* was obtained in the control (#1) and hybrid (#2) but not in the *G. volans* control (#16) while for *Cytb* a 104-bp band specific for *G. sabrinus* was also present in the control (#1) and hybrid (#2) but not in the *G. volans* control (#16).

A total of 36 Southern Flying Squirrels (19 females, 17 males) were captured in 2017 and 33 new (19 females, 14 males) and 3 (all female) previously tagged squirrels were captured in 2018. In total, 9 individuals (2 females, 7 males) were used for respirometry experiments in 2017 and 12 (7 females, 5 males) were used in 2018. Mass of the adults captured varied from 49.1 to 76.5 g (mean 63.3 ± 6.6, *n* = 52) at first capture (adults and nonreproductive females), and 49.1 to 79.2 g (mean 60.6 ± 8.1, *n* = 21) for individuals used in the respirometry experiments. Two male flying squirrels in 2017 and 5 males in 2018 were implanted with data loggers, with 1 data logger recovered in 2017 and 4 in 2018 (Supplementary Data SD4). Genotyping assays performed on tissue samples collected from all individuals in this study using multiplex PCR markers based on a mitochondrial and a nuclear gene (Rogic et al. 2016) were consistent with *G. volans* for all samples tested (Fig. 1).

**Fig. 2.**
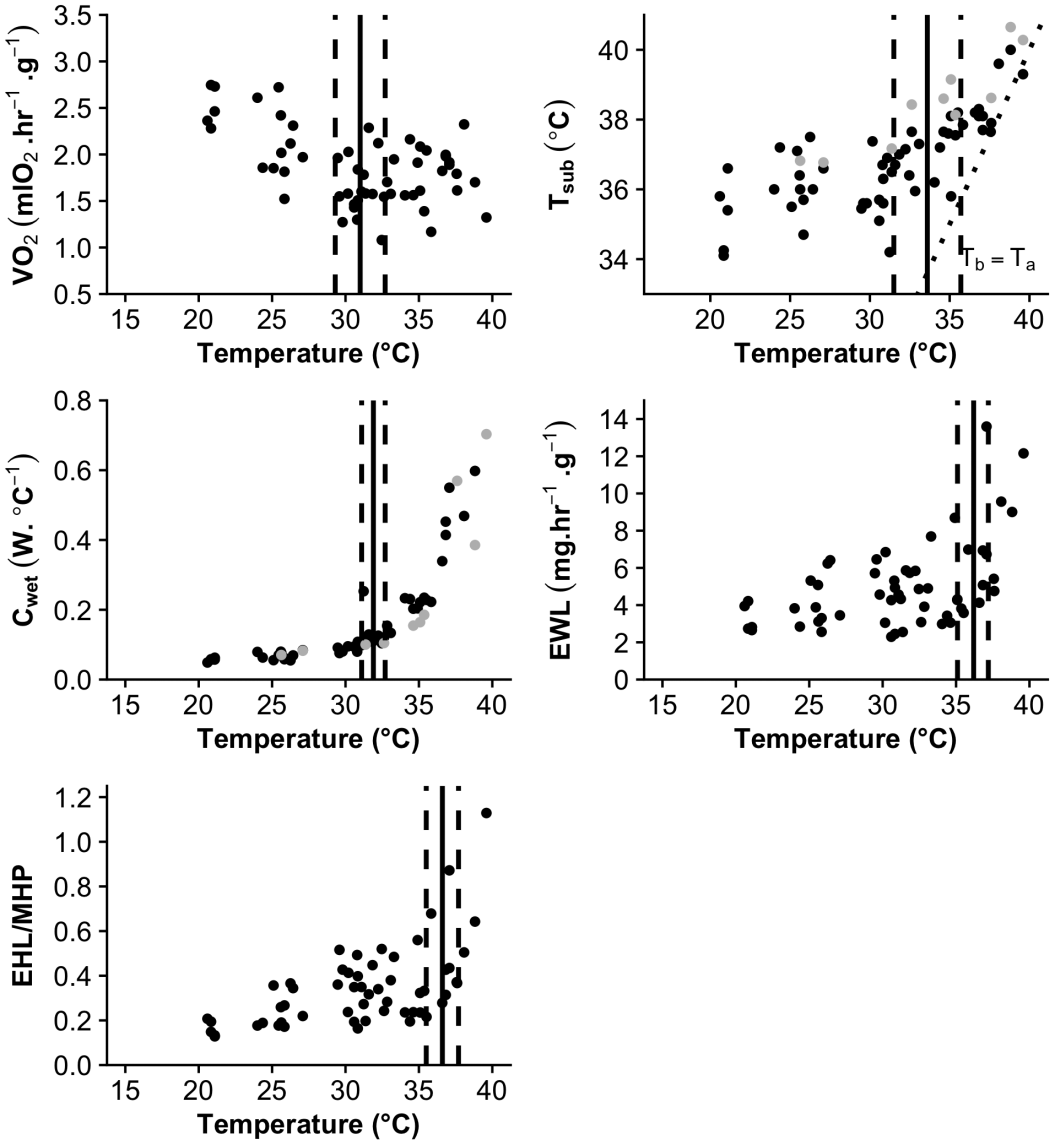
Physiological parameters of *Glaucomys volans* exposed to various ambient temperatures during flow-through respirometry experiments. Inflection points, calculated via break point analyses in the data are drawn as solid black lines with 95% CIs indicated by dashed lines. The dotted line indicates equality between subcutaneous body temperature (*T*_sub_) and ambient temperature (*T*_a_).

A total of 53 resting metabolic rates spanning 20 to 40 °C were collected from 21 squirrels (~2 to 4 per individual). The best-fitting model for estimating resting metabolic rate included ambient temperature and mass as predictor variables (Supplementary Data SD5). An inflection point was found in the relationship between the rate of oxygen consumption and ambient temperature, indicating that the lower critical limit of thermoneutrality was 29.8 °C (95% CI: 27.5 to 32.1 °C; Fig. 2). There was no inflection point in the rate of oxygen consumption at a higher temperature which would have indicated the upper critical limit of thermoneutrality. However, using the same best-fitting model, inflection points were determined for evaporative water loss (EWL), subcutaneous temperature (*T*_sub_), and the ratio of evaporative heat loss to metabolic heat production (EHL/MHP). The breakpoints and their 95% CIs are: EWL = 36.2 °C (35.2 to 37.3 °C); *T*_sub_ = 33.64 °C (31.5 to 35.7 °C); and EHL/MHP = 36.58 °C (35.5 to 37.7 °C; Fig. 2). Three individuals had simultaneous measurements of both subcutaneous body temperatures and core body temperatures. Although the trend in both was the same (decreasing with decreasing ambient temperatures) subcutaneous temperatures consistently underestimated core body temperature (mean −0.72 °C ± 0.25; Supplementary Data SD6).

**Fig. 3.**
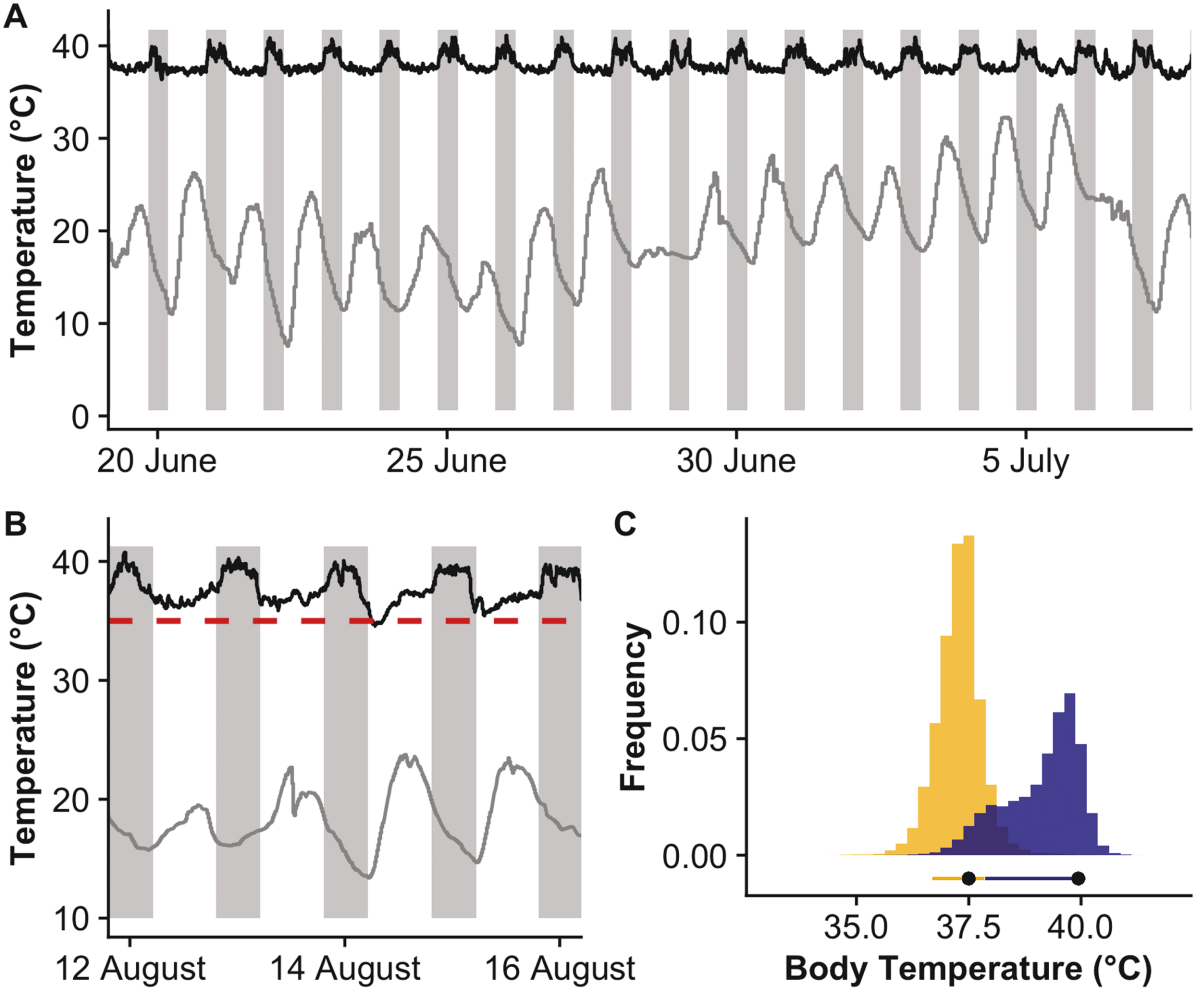
Free-ranging body temperatures of Southern Flying Squirrels (*Glaucomys volans*), black lines represent core body temperature and gray line shows ambient temperature of Dwight B. Demeritt Forest South. (A) A multiday trace from a male showing the day with highest daytime (rest phase) body temperature (5 July). (B) A few days surrounding a possible shallow torpor bout (*T*_b_ < 35 °C) in a male Southern Flying Squirrel (UM031)—body temperature dropped to 34.6 °C for a few hours before increasing as ambient temperature increased, the dashed line indicates 35 °C. (C) A frequency histogram of all body temperatures measured from all 5 individuals in the study. Daytime rest-phase temperatures (determined by sunrise/sunset times at the study site) are illustrated in yellow and the warmer nighttime active-phase temperatures in blue. The bars below histograms indicate the limits of the 10th and 90th quantiles of body temperature distribution for each phase with black dots representing the mode.

Free-ranging flying squirrels showed a clear pattern of body temperature fluctuation matching their daily activity cycle, with higher body temperature during the active nighttime phase and a lower body temperature during the resting daytime phase—the modal *T*_b_ for active squirrels is 39.9 °C and for resting squirrels is 37.5 °C (Fig. 3; Table 1). The average Heterothermy Index ranged from 1.9 to 2.2 and the average daily amplitude identified using nonstationary waveform analysis (following Levesque et al. 2017) ranged from 3.3 to 4.1 °C (Table 1). No instances of torpor were observed although 1 individual (UM031) had a possible shallow torpor bout with body temperature dropping below 35 °C for an hour (Fig. 3B). Model testing revealed the significant driver of average maximum daily body temperature to be day of the year, with higher body temperatures observed earlier in the summer although the impacts were minimal (0.0022 °C/day). The average maximum ambient temperature was an additional driver in comparably strong models with higher body temperatures being observed on warmer days (Supplementary Data SD5; Fig. 2). Both day of the year and average minimum ambient temperature were significant predictor variables of average minimum body temperature in the best-fitting model (Supplementary Data SD5). Similar to average maximum body temperature, the average body temperature range was best predicted by day of the year, with ambient temperature range being a significant variable in comparable models (Table 1).

**Table 1.** Means ± standard deviations of various parameters of body temperature of free-ranging Southern Flying Squirrels (*Glaucomys volans*) in Maine.

	UM031		UM073		UM076		UM726		UM802	
	*n* = 103		*n* = 90		*n* = 64		*n* = 97		*n* = 71	
	Day	Night	Day	Night	Day	Night	Day	Night	Day	Night
Mean *T*_b_ (°C)										
Mean	37.3 ± 0.3	39.1 ± 0.4	37.2 ± 0.4	39.0 ± 0.3	37.3 ± 0.3	39.4 ± 0.2	37.3 ± 0.3	39.0 ± 0.3	37.5 ± 0.3	39.3 ± 0.3
Range	35.8 − 37.8	37.7 − 39.7	36.6 − 38.8	38.2 − 39.5	36.6 − 37.9	38.9 − 39.7	36.5 − 38.1	37.9 − 39.7	36.5 − 38.2	38 − 39.9
SD *T*_b_ (°C)										
Mean	0.3 ± 0.2	0.8 ± 0.2	0.3 ± 0.1	0.8 ± 0.1	0.3 ± 0.1	0.7 ± 0.1	0.4 ± 0.1	0.7 ± 0.2	0.3 ± 0.1	0.7 ± 0.1
Range	0.1 – 1	0.1 − 1.2	0.2 − 1	0.5 − 1.1	0.2 − 0.6	0.5 – 1	0.2 − 1.1	0.4 − 1.1	0.2 − 0.8	0.5 − 0.8
Mode *T*_b_ (°C)										
Mean	37.2 ± 0.4	39.4 ± 0.8	37.2 ± 0.4	39.3 ± 0.7	37.3 ± 0.3	39.8 ± 0.3	37.2 ± 0.4	39.1 ± 0.7	37.5 ± 0.3	39.6 ± 0.5
Range	35.4 – 37.8	36.9 – 40.2	36.1 – 38.6	37.2 – 40	36.4 – 38	38.6 – 40.3	36.1 – 37.9	37.5 – 39.9	36.5 – 38.2	37.7 – 40.2
Min *T*_b_ (°C)										
Mean	36.7 ± 0.5		36.4 ± 0.5		36.6 ± 0.4		36.4 ± 0.4		36.8 ± 0.3	
Range	34.6 – 37.3		35.1 – 38		35.7 – 37.4		35.6 – 37.1		35.9 – 37.4	
Max *T*_b_ (°C)										
Mean	40.2 ± 0.3		40.2 ± 0.3		40.4 ± 0.4		40.6 ± 0.6		40.2 ± 0.4	
Range	37.9 – 40.8		38.6 – 40.7		37.6 – 41		37.7 – 41.2		37.7 – 40.9	
Max *T*_b_ − Min *T*_b_ (°C)										
Mean	3.6 ± 0.6		3.8 ± 0.6		3.9 ± 0.5		4.2 ± 0.7		3.5 ± 0.5	
Range	0.8 – 5.6		0.6 – 5.1		1.9 – 4.8		1.2 – 5.3		1.2 – 4.5	
Heterothermy Index										
Mean	1.9 ± 0.3		1.9 ± 0.3		2.2 ± 0.2		1.8 ± 0.3		1.9 ± 0.2	
Range	1.5 – 2.9		0.7 – 2.4		1.7 – 2.7		1.3 – 2.5		1.4 – 2.6	
Additive quantile regression wavelet analysis										
Period (days)	1 ± 0.7		0.9 ± 0.2		1.1 ± 0.3		1 ± 0.6		0.9 ± 0.3	
Amplitude (°C)	3.5 ± 0.6		3.8 ± 0.6		4 ± 0.3		4.1 ± 0.7		3.3 ± 0.6	

## Discussion

Our study presents the first data on responses of free-ranging members of the Sciurid subfamily Pteromini (flying squirrels) to high temperatures. Flying squirrels are an unusual group in that the larger species (>1 kg) are found in subtropical and tropical regions while the smaller species (<300 g) are found in temperate and even polar regions (Rocha et al. 2016). This pattern runs contrary to most zoogeographic patterns, where generally the larger species are found toward the poles (Meiri and Dayan 2003). It should be noted, however, that the pattern does hold within the genus *Glaucomys* and the southern species measured in this study is smaller (~60 g) than the northern species (~94 to 107 g; Olson et al. 2017). We found that Southern Flying Squirrels do not significantly increase their resting metabolic rate with increasing ambient temperature, but they do show an increase in evaporative water loss and body temperature at temperatures above ~35 °C. The flexibility in resting body temperatures measured during respirometry experiments was mirrored in the free-ranging body temperatures where minimum daily body temperatures were impacted by environmental temperatures. Combined, these findings indicate that Southern Flying Squirrels, at least at the northern edge of their range, will not have to alter energy budgets to keep up with increasing temperatures. This is perhaps not surprising given that their range extends down through central America (Cassola 2016a). It would therefore be of interest to use similar assessments of thermal tolerance at the southern edge of the range, in Central America, to fully determine the capacity of the species to deal with high temperatures as well as perform a similar assessment for the Northern Flying Squirrel throughout its range.

Even at the northern edge of its geographical distribution, the Southern Flying Squirrel has a relatively high thermal tolerance for a nocturnal small mammal. The estimated lower critical temperature of 29.8 °C falls within the range of estimations from previous studies (26.9 to 35 °C; Neumann 1967; Stapp 1992; Olson et al. 2017). At the warmer end of our sampling range (~40 °C) we did not detect an inflection point in metabolic rate that would indicate the upper limit of thermoneutrality. This is in contrast to Neumann (1967) who found an increase in metabolic rate at 36 °C in summer-acclimated individuals. However, since the Neumann (1967) study monitored metabolic rate and body temperature but not activity or evaporative water loss, it is unclear if the increase observed at the higher temperatures was due to metabolism alone or other factors such as increases in activity. Recent research has also questioned the utility of diagnosing upper limits in thermoneutrality using increases in metabolism alone, as not all means of evaporative cooling are energetically costly (Mitchell et al. 2018; McKechnie and Wolf 2019; Thonis et al. 2020; Levesque and Marshall 2021). Thus, diagnosing tolerance to heat needs to include measures of evaporative cooling as well as test for the potential to store heat by increasing body temperatures along with ambient temperature (Mitchell et al. 2018; Gerson et al. 2019; Thonis et al. 2020). Although we did not detect an increase in metabolism at higher ambient temperatures, we did record an increase in evaporative water loss at 36.2 °C which was accompanied by postural changes and increased body temperature. As some authorities consider increases in evaporative cooling to be indicative of the upper limits of thermoneutrality (IUPS Thermal Commission 2003; Withers et al. 2016; Mitchell et al. 2018), we could realistically consider the thermoneutral zone of Southern Flying Squirrels in Maine to be from 29.8 to 36.2 °C. While the number of extremely hot and humid days is expected to increase with climate change, the state of Maine is far less likely than other parts of the world to see temperatures above 36 °C, and even the southernmost portions of the state are predicted to only see ~15 of these days by the 2050s (Fernandez et al. 2020). Unless coincident with widespread disease or a severe drop in resources, most flying squirrel individuals can likely compensate for the higher energy usage needed to cool themselves for 2 weeks of extremely hot weather.

Thermal tolerance assessed in the laboratory during the rest phase of an animal has limited ecological relevance for assessing the response of a species to climate (Mitchell et al. 2018; Levesque and Marshall 2021). However, we argue that for nocturnal species—who rest during the warmer parts of the day—it can provide adequate estimates of the potential costs of raising temperatures, especially for arboreal species whose rest sites might be more exposed to temperature variability than ground nesting species (Lovegrove et al. 2014; Youngentob et al. 2021). Subcutaneous and body temperatures increased with increasing ambient temperature in the respirometry experiments (Fig. 2). As resting body temperatures in small mammals are known to correlate with ambient temperatures (Refinetti 1997), and rest-phase increases in body temperature have been observed in a number of nocturnal arboreal mammals (Lovegrove et al. 2014; Welman et al. 2017; Dausmann et al. 2023), we used free-ranging body temperatures measured over the warm summer months to look at potential effects of high ambient temperature on flying squirrel energetics. We found that Southern Flying Squirrels have a higher body temperature at night during their active phase and lower during the day when resting (Fig. 3; Table 1). Similar to previous laboratory studies on this species (Refinetti 1998, 1999), the daily rhythmicity in body temperature closely matched photoperiod. The standardized Heterothermy Index (Boyles et al. 2011) allows for comparison of body temperature variability both within and between species. The average Heterothermy Index value for all squirrels in this study was 1.93 (Table 1), which is close to those calculated for 2 other Sciurids—the Arctic Ground Squirrel (*Urocitellus perryii*, HI = 1.43) and Cape Ground Squirrel (*Xerus inauris*, HI = 1.89)—yet greater than all other rodents with a published HI (minimum HI = 0.70, maximum HI = 1.36; Boyles et al. 2013). Similarly, the average daily difference between resting and active body temperatures—which has more available comparative data—in our study (3.8 °C ± 0.6) is also on the high side for small mammals, e.g., ~3 °C measured in the diurnal ground nesting White-tailed Antelope Squirrel (*Ammospermophilus leucurus*; Refinetti and Kenagy 2018, 2023) but lower than the nocturnal arboreal Black-tailed Tree Rat (*Thallomys nigricauda*) which ranged from 3 °C in the summer to >7 °C in the winter (Coleman and Downs 2010). We currently lack a comprehensive, comparative data set of mammalian body temperature rhythms. However, the difference between the modal active (39.4 °C) and resting (37.3 °C) body temperatures observed in this study (2.1 °C) was higher than most of the 14 species analyzed in a recent review (Levesque et al. 2021). What is perhaps more surprising is that both the resting and active modal temperatures are in the upper percentiles for mammals with the resting temperatures near the 70th percentile and active temperatures falling above the 80th percentile in the range of supraendotherms (sensu Lovegrove 2012).

Flying squirrels (tribe Pteromyini) are unique within the Family Sciuridae in having shifted from the ancestral Sciurid condition of a diurnal lifestyle to being nocturnal (Carvalho et al. 2006; Bennie et al. 2014). The Pteromyini, along with the Holartic tree squirrels (tribe Sciurini), form the Sciurinae subfamily and appear to share some of their physiological traits. The Sciurini have higher basal metabolism and body temperatures than Holartic ground squirrels (tribe Marmotini), despite inhabiting similar environments (Clarke and O’Connor 2014; Genoud et al. 2017), while also remaining homeothermic (Brigham and Geiser 2012; Dausmann et al. 2013). Flying squirrels have, to date, generally been found to be homeothermic and their basal metabolism is closer to that of the tree squirrels than the ground squirrels (Merritt et al. 2001). However, they have anecdotally been shown to use torpor in the field (Muul 1968) and shallow torpor has been induced in the laboratory (Olson et al. 2017). Our findings of high and relatively constant body temperatures throughout the study confirm the characterization of closer physiological alignment of flying squirrels to Holarctic tree squirrels than ground squirrels. Although the shallow dips in body temperature below 36 °C observed in a few cases fit the definition of shallow torpor (Nowack et al. 2023), they were only rarely observed. To gain a full understanding of torpor use in flying squirrels more data would be needed from the winter months.

The variability in the body temperature of Southern Flying Squirrels was driven in part by ambient temperature, but not to the degree we had anticipated. Flying squirrels, like other small mammals, inhabit their own microclimate which may greatly differ from the average forest temperature (Varner and Dearing 2014). Flying squirrels construct nests in tree hollows or on the forest floor using various plant fibers, but the insulative effect of nests has been studied primarily in cold rather than weather (Muul 1968, 1974; Trudeau et al. 2011; Zweep et al. 2018). In the winter, tree hollow nests sustain warmer temperatures and reduced variability, resulting in energy savings for flying squirrels (Muul 1968). Whether or not flying squirrels are reaping thermal benefits from nest sites in the summer is currently unknown. The research of Stains (1961) and Isaac et al. (2008) suggests that nest temperature should be less variable than overall forest temperature and that cooler hollows may be preferred. However, without direct measurements of nest temperature and occupancy, it is impossible to assert that nest sites confer a thermal benefit to flying squirrels in summer. Furthermore, insulative properties of the nest itself are not the only factors determining the microclimate for a flying squirrel. Group nesting is well documented in all species of North American flying squirrel (Muul 1974; Dolan and Carter 1977; Wells-Gosling and Heaney 1984) and is primarily used to maintain warmth in the winter and reduce the energy required for thermoregulation. However, flying squirrel aggregations are found year-round throughout the geographic range of *G. volans* and *G. sabrinus* (Muul 1974; Stapp 1992; Layne and Raymond 1994). Large aggregations of flying squirrels increase temperature within a nest through thermoregulation, increase insulation of the nest with additional pelage, and decrease heat loss by decreasing the surface area to volume ratio (Muul 1968). Despite being a benefit in the winter, group nesting could be a detriment as temperatures rise (Muul 1974). Temperatures of group nests could get too warm, forcing flying squirrels to change behavior. Such changes could include increased use of cooling mechanisms via higher energy expenditure, solitary nesting, or producing smaller litters to reduce maternal heat loads. Regardless of the coping mechanism, flying squirrels will need to find some way to remedy the high heat load generated from their current nesting behaviors. Furthermore, the individuals in our study were either males or nonreproductive females, and the high heat loads placed on females during gestation and lactation (Krol et al. 2003; Guillemette et al. 2009) are also in need of further investigation.

Southern Flying Squirrels showed a high level of thermal tolerance with metabolic rate remaining at basal levels even at the highest ambient temperatures measured (~41 °C) and did not significantly increase rates of evaporative heat loss until above 36 °C. The respirometry data were mirrored by field data where diurnal rest-phase body temperatures were only slightly impacted by ambient temperatures during both rest and the previous activity period. These findings indicate that the southern species, at least at the northern edge of its geographic range, is not likely to be impacted by raising environmental temperatures. Despite resting in nest sites vulnerable to high ambient temperatures during the day (tree holes), southern flying squirrels do not significantly increase their resting metabolic rate with increasing ambient temperature, but they do show an increase in evaporative water loss and body temperature. Incidence and degree of heterothermy may also change as ambient temperatures increase. The free-ranging body temperature data revealed a level of heterothermy on par with other squirrel species, including one living in a hot, arid environment. The experimental period was too short, however, to reveal any seasonal changes and did not provide an indication of costs that may be associated with the observed level of heterothermy. More research on the seasonality of and constraints to heterothermy needs to be conducted to better understand how this thermal strategy fits into the ecology of flying squirrels. Similarly, Northern Flying Squirrels may show a marked increase in resting metabolic rate as temperatures rise or initiate evaporative cooling at a lower ambient temperature. Additional research on the microclimate experienced by flying squirrels is crucial for improved understanding of the results of this study and for the accurate prediction of future changes in flying squirrel distribution in North America.

## Supplementary data

Supplementary data are available at *Journal of Mammalogy* online.

**Supplementary Data SD1.**—Numbering system for the flying squirrel samples to be genotyped using PCR and gel electrophoresis. Each sample corresponds to the trapping site that the squirrels were trapped at during the summer of 2017.

**Supplementary Data SD2.**—Primers designed by Rogic et al. (2016) to genotype *Glaucomys sabrinus* and *G. volans*. The listed primer sets show the inclusion of the forward primer, the reverse primer, and the species-specific primer designed for each species.

**Supplementary Data SD3.**—Additional methods for the respirometry experiments and the surgical methods.

**Supplementary Data SD4.**—Information on the start weight, date of temperature-sensitive data logger implantation, and retrieval of male southern flying squirrels.

**Supplementary Data SD5.**—Rank of linear models evaluating the effects of various factors on the resting metabolic rates and body temperatures of *Glaucomys volans*.

**Supplementary Data SD6.**—Core body temperature (measured via implanted temperature-sensitive data loggers) as a function of subcutaneous body temperature (obtained from PIT tags injected interscapularly) in southern flying squirrels (*Glaucomys volans*, *n* = 3) measured during flow-through respirometry experiments.

gyae041_suppl_Supplementary_Datas_SD1

gyae041_suppl_Supplementary_Datas_SD2

gyae041_suppl_Supplementary_Datas_SD3

gyae041_suppl_Supplementary_Datas_SD4

gyae041_suppl_Supplementary_Datas_SD5

gyae041_suppl_Supplementary_Datas_SD6
