# Supplementary material for: Assessing responses to heat in a range-shifting, nocturnal, flying squirrel
Source: J Mammal. 2024 May 11;105(4):899–909. doi: 10.1093/jmammal/gyae041 (PMC11285193; doi:10.1093/jmammal/gyae041)
Supplement: gyae041_suppl_Supplementary_Datas_SD1 [file gyae041_suppl_supplementary_datas_sd1.docx]

**Supplementary data (SD1): Assessing responses to heat in a range shifting nocturnal arboreal small mammal**

Hensley et al. 2023

Numbering system for the flying squirrel samples to be genotyped using PCR and gel electrophoresis. Each sample corresponds to the trapping site that the squirrels were trapped at during the summer of 2017.

| Sample Number | Trapping Location |
| --- | --- |
| #1 | Northern Maine (*G. sabrinus* control sample) |
| #2 | N/A – artificial hybrid |
| #3 | DeMerrit Forest |
| #4 | DeMerrit Forest |
| #5 | DeMerrit Forest |
| #6 | DeMerrit Forest |
| #7 | DeMerrit Forest |
| #8 | DeMerrit Forest |
| #9 | UM campus |
| #10 | UM campus |
| #11 | UM campus |
| #12 | UM campus |
| #13 | UM campus |
| #14 | UM campus |
| #15 | UM campus |
| #16 | Holt Forest (*G. volans* control) |
| #17 | Unknown |
| #18 | Unknown |
| #19 | Unknown |
