# Supplementary material for: Assessing responses to heat in a range-shifting, nocturnal, flying squirrel
Source: J Mammal. 2024 May 11;105(4):899–909. doi: 10.1093/jmammal/gyae041 (PMC11285193; doi:10.1093/jmammal/gyae041)
Supplement: gyae041_suppl_Supplementary_Datas_SD2 [file gyae041_suppl_supplementary_datas_sd2.docx]

**Supplementary data (SD2): Assessing responses to heat in a range shifting nocturnal arboreal small mammal**

Hensley et al. 2023

Primers designed by Rogic et al. (2016) to genotype *Glaucomys sabrinus* and *Glaucomys volans*. The listed primer sets show the inclusion of the forward primer, the reverse primer, and the species-specific primer designed for each species.

| **Cytb Primer Mix** | **CNR1 Primer Mix** |
| --- | --- |
| Cytb L14724  5’ – CGAAGCTTGATATGAAAAACCAT  CGTTG – 3’ | CNR1-5R  5’–CAGACTGMAGCTTCTTGCAGTTCC-3’ |
| Cytb H15149  5’ – AAACTGCAGCCCCTCAGA  ATGATATTTGTCCTCA – 3’ | CNR1-11F  5’ – AGTGTGGGGAGAACTTC  ATGGACAT – 3’ |
| Cytb-*sabrinus*-2F  5’ – NNCGCAAATGGTGCT – 3’ | CNR1-*sabrinus-*R  5’ – GCTCCCCAGAAGGTCTGCC – 3’ |
