# Supplementary material for: Assessing responses to heat in a range-shifting, nocturnal, flying squirrel
Source: J Mammal. 2024 May 11;105(4):899–909. doi: 10.1093/jmammal/gyae041 (PMC11285193; doi:10.1093/jmammal/gyae041)
Supplement: gyae041_suppl_Supplementary_Datas_SD3 [file gyae041_suppl_supplementary_datas_sd3.docx]

**Supplementary data (SD3): Assessing responses to heat in a range shifting nocturnal arboreal small mammal**

Hensley et al 2023

**Supplementary methods for respirometry**

The metabolic chamber was a 1.9L airtight, plastic container (Lock and Lock Co., Seoul, South Korea) with 3 ports (Compression Push Fitting Adapters, John Guest, USA): one for the incurrent airstream, one for the excurrent airstream, and one for a thermocouple. A mesh wire grate kept the squirrel elevated above the temperature sensor, the inflow ports and mineral oil (99.99% or higher) which was placed in the bottom of the container to absorb any urine or feces produced by the animal. Temperature inside the metabolic chamber was monitored using a thermocouple and meter (TC-2000 Type-T Thermocouple Meter, Sable Systems, North Las Vegas, NV) and recorded with a temperature data logger (DS1922L Thermochron iButtons, Maxim Integrated, San Jose, CA) attached to the chamber base and lid. Subcutaneous temperature of the squirrel was monitored via a pit-tag and recorded using a Biomark HPR Plus Reader (Biomark Inc., Boise, ID). Prior to the study we tested a subset of the transponders in a water bath over the range of expected body temperatures (30-45°C) and compared to the nearest 0.1°C using a glass mercury thermometer (traceable to a national standard) and found no significant differences.

The metabolic chamber was placed in either a temperature-controlled cabinet (Incubator, Refrigerated with Mechanical Convection, KB 53, BINDER, Long Island, New York) or cooler box (Pelt-5, Sable Systems, North Las Vegas, NV) depending upon equipment availability. The incurrent airstream was pulled through a column containing the desiccant Drierite (W. A. Hammond Drierite Co. Ltd., Xenia, OH) by a 1.0 L/min pump. Once dried, the incurrent air was divided into two airstreams – one for the chamber and one for the control stream. Factory calibrated gas mass flow controllers (MC-Gas Mass Flow Controllers, Alicat Scientific, Tucson, AZ) regulated the incurrent airflow to within 0.8% of the desired flow rate. The chamber flow rate ranged between 500-1650 ml/min., increasing as ambient temperature increased to maintain a stable chamber humidity (15 ppt H_2_O). Air was pushed through a coil of copper tubing to equilibrate its temperature to that of the cabinet and then into the chamber. Air from the respirometer entered a gas flow switcher (BL-2 Baselining System or RM-8 Flow Multiplexer, Sable Systems, North Las Vegas, NV) which switched between the control stream for 5 minutes and the chamber stream for 40 minutes. The sampled air stream was pulled through the gas analyzers, carbon dioxide then oxygen, via a second pump (SS-4 Subsampler, Sable Systems, North Las Vegas, NV) before finally exiting the system. A combined water and carbon dioxide gas analyzer was used for every experiment (LI-840A CO_2_/H_2_O Gas Analyzer, LI-COR, Lincoln, NE). Additionally, an oxygen analyzer (FC-10 Oxygen Analyzer, Sable Systems, North Las Vegas, NV) was used whenever possible but was not available for all experiments. O_2_, CO_2_, water vapor, and analyzer cell pressure measurements were recorded every second using ExpeData-P Analysis Software (Sable Systems, North Las Vegas, NV) and LI-840A Software (LI-COR, Lincoln, NE). The LI-840A was calibrated using pure nitrogen as a zero (Matheson Tri-Gas, Basking Ridge, NJ), a 1.95% CO_2_ certified span gas (Matheson Tri-Gas, Basking Ridge, NJ) and the water-vapor span values were set by generating humid air of a standard dew point using a water bath and a bubbler flask. Additionally, an infrared USB camera recorded the squirrel’s activity and allowed for non-disruptive monitoring during the experiment.

Time lag between analyzers was negligible (<1s), therefore measurements of flow rate, chamber temperature, subcutaneous temperature, and oxygen (if applicable), were matched to each 5-minute period of low CO_2_. All measurements of oxygen consumption and carbon dioxide production were transformed into $\dot{V}$O_2_ and $\dot{V}$CO_2_ using equations from Lighton (2008). The respiratory quotient (RQ),$\dot{V}$CO_2_/$\dot{V}$O_2_ was calculated for every selected 5-minute period in a single experiment and then averaged across all periods and all trials that included the oxygen analyzer to generate a single RQ value for flying squirrels, 0.7, which was used to solve for $\dot{V}$O_2_ and $\dot{V}$CO_2_ for experiments run without the oxygen analyzer. Resting metabolic rate (RMR) was converted to metabolic rate (W) using an oxycaloric equivalence calculated from the respiratory quotient (6.0913*RQ+15.439 J. m$l$O_2_ ^-1^). Water vapor concentration was transformed into water vapor density (WVD), evaporative water loss (EWL), and, ultimately, evaporative heat loss (EHL) and a latent heat of vaporization value of T_a_*(-2.39)+2498.42 J.mgH_2_O^-1^(Withers 1992).

Surgical methods

All loggers were sterilized in Isopropyl alcohol for a minimum of 4 hours prior to implantation. The squirrels were given ~0.01g of Capofran analgesic delivered on a slice of apple 2 hours before and immediately after surgery to minimize pain and were weighed to the nearest gram before and after each surgery. Squirrels were placed in an air-tight, plastic container for sedation via vaporized Isoflurane (Isoflurane, USP, Piramal Healthcare, Inc., Bethlehem, PA). A 5% concentration of Isoflurane was administered at a rate of 700-800 ml/min until the squirrel could no longer right itself and appeared to be sedated. The squirrel was then removed from the chamber and a small mask applied to its face to continue administering Isoflurane at a 2-3% concentration for the duration of the surgery. The ventral surface was prepped for incision by shaving a 3 cm x 5 cm section of fur and cleaned with a betadine scrub, isopropyl alcohol, and a betadine solution (Betadine Surgical Scrub (povidone-iodine, 7.5%) and Betadine Solution (povidone-iodine, 10%), Purdue Pharma L.P., Stamford, CT). Once cleaned, a small incision was made, not exceeding 2 cm, in the skin and abdominal muscle tissue along the linea alba. The temperature sensitive data logger was placed inside the intraperitoneal cavity and the incision was sealed with dissolvable sutures (Coated VICRYL (Polyglactin 910) Suture, Ethicon, Inc., Bridgewater, NJ) and tissue glue (Vetbond Tissue Adhesive, 3M, St. Paul, MN). An antibacterial ointment (Neosporin Antibacterial Ointment, Johnson & Johnson Consumer Inc., New Brunswick, NJ) was applied to the surgical site to prevent infection.

**Literature cited**

Lighton, J. R. B. 2008. Measuring Metabolic Rates: A Manual for Scientists. Oxford University Press, USA.

Withers, P. C. 1992. Comparative Animal Physiology. Saunders College Pub., Fort Worth.
