# Supplementary material for: Assessing responses to heat in a range-shifting, nocturnal, flying squirrel
Source: J Mammal. 2024 May 11;105(4):899–909. doi: 10.1093/jmammal/gyae041 (PMC11285193; doi:10.1093/jmammal/gyae041)
Supplement: gyae041_suppl_Supplementary_Datas_SD4 [file gyae041_suppl_supplementary_datas_sd4.docx]

**Supplementary data (SD4): Assessing responses to heat in a range shifting nocturnal arboreal small mammal**

Hensley et al. 2023

Information on the start weight, date of temperature-sensitive data logger implantation and retrieval of male southern flying squirrels. *Recovered from deceased individual.

| **Squirrel ID** | **Mass (g)** | **Implantation Date** | **Retrieval Date** | **Total Days Deployed** |
| --- | --- | --- | --- | --- |
| UM031 | 62 | 4 Aug. 2017 | 15 Nov. 2017 | 103 |
| UM073 | 65 | 8 May 2018 | 6 Aug. 2018 | 90 |
| UM076 | 63 | 9 June 2018 | 12 Aug. 2018 | 64 |
| UM726 | 61 | 8 May 2018 | 13 Aug. 2018* | 97 |
| UM802 | 63 | 22 June 2018 | 1 Sept. 2018 | 71 |
