# Supplementary material for: Assessing responses to heat in a range-shifting, nocturnal, flying squirrel
Source: J Mammal. 2024 May 11;105(4):899–909. doi: 10.1093/jmammal/gyae041 (PMC11285193; doi:10.1093/jmammal/gyae041)
Supplement: gyae041_suppl_Supplementary_Datas_SD5 [file gyae041_suppl_supplementary_datas_sd5.docx]

**Supplementary data (SD5): Assessing responses to heat in a range shifting nocturnal arboreal small mammal**

Hensley et al. 2023

Rank of linear models evaluating the effects of various factors on the resting metabolic rate of *Glaucomys volans*. Ranking was performed using corrected Akaike Information Criterion (AICc) scores and Akaike weights (AICcWt). All models with an AICcWt > 0 are presented and the number of parameters contained in the model are included (k). Breakpoints in EWL, T_sub_ an MHP/EHL could not be included in the models due to small samples sizes at T_a_s above the breakpoint.

| **Model Parameters** | **k** | **AIC_c_** | **AIC_c_Wt** |
| --- | --- | --- | --- |
| RMR^b^ |  |  |  |
| ‘T_a_<29.8’ + ‘T_a_>29.8’ + ‘StartMass’ | 6 | 513.39 | 0.75 |
| ‘T_a_<29.8’ + ‘T_a_>29.8’+ ‘StartMass’+ ‘Sex’ | 7 | 515.75 | 0.23 |
| ‘T_a_<29.8’ + ‘T_a_>29.8’ | 5 | 521.45 | 0.01 |
| ‘T_a_’ | 4 | 525.48 | 0.00 |
|  |  |  |  |
| EWL^a^ |  |  |  |
| ‘T_a_<36.2’ | 3 | -131.24 | 0.50 |
| ‘T_a_’ | 3 | -129.15 | 0.18 |
| ‘T_a_<36.2’ + ‘StartMass’ | 4 | -129.13 | 0.18 |
| ‘+ ‘StartMass’+ ‘Sex’ | 5 | -128.76 | 0.15 |
|  |  |  |  |
| T_sub_^b^ |  |  |  |
| ‘T_a_’ + ‘StartMass’ | 5 | 127.50 | 0.69 |
| ‘T_a_’ + ‘StartMass’ + ‘Sex’ | 6 | 129.11 | 0.31 |
| ‘T_a_’ | 4 | 155.73 | 0.00 |
|  |  |  |  |
| MHP/EHL^a^ |  |  |  |
| ‘T_a_’ | 3 | -70.10 | 0.61 |
| ‘T_a_’ + ‘StartMass’ | 4 | -68.58 | 0.28 |
| ‘T_a_’ + ‘StartMass’ + ‘Sex’ | 5 | -66.64 | 0.11 |
| T_a_<36.6’ | 3 | -89.36 | 0.0 |

^a^Variance structure used: weights = varFixed(~Ta), ^b^Variance structure used: weights = varIdent(form=~1|Sex)

Rank of linear mixed models evaluating the effects of various factors on the body temperature (T_b_) of free-ranging *Glaucomys volans*. Ranking was performed using corrected Akaike Information Criterion (AICc) scores and Akaike weights (AICcWt). All models with an AICcWt > 0 are presented and the number of parameters contained in the model are included (k).

| **Model Parameters^a,b^** | **k** | **AIC_c_** | **AIC_c_Wt** |
| --- | --- | --- | --- |
| T_bmax_ |  |  |  |
| ‘Date’ + ‘MaxT_a_’ | 6 | -103.50 | 0.44 |
| ‘Date’ | 5 | -103.26 | 0.39 |
| ‘Date’ * ‘MaxT_a_’ | 7 | -101.55 | 0.17 |
| ‘MaxT_a_’ | 5 | -90.97 | 0.00 |
|  |  |  |  |
| T_bmin_ |  |  |  |
| ‘Date’ * ‘MinT_a_’ | 7 | 211.02 | 0.72 |
| ‘Date’ + ‘MinT_a_’ | 6 | 212.90 | 0.28 |
| ‘MinT_a_’ | 5 | 221.16 | 0.00 |
| ‘Date’ | 5 | 242.20 | 0.00 |
|  |  |  |  |
| T_bdelta_ |  |  |  |
| ‘Date’ | 7 | 309.66 | 0.49 |
| ‘Date’ + ‘deltaT_a_’ | 8 | 311.09 | 0.24 |
| ‘deltaT_a_’ | 7 | 311.97 | 0.15 |
| ‘Date’ * ‘deltaT_a_’ | 9 | 312.39 | 0.12 |

**^a^All models were corrected for autocorrelation using the correlation structure corCAR1 (form = ~1|Animal ID, ^b^~1|Animal ID was used as a random factor.**
