# Supplementary material for: Assessing responses to heat in a range-shifting, nocturnal, flying squirrel
Source: J Mammal. 2024 May 11;105(4):899–909. doi: 10.1093/jmammal/gyae041 (PMC11285193; doi:10.1093/jmammal/gyae041)
Supplement: gyae041_suppl_Supplementary_Datas_SD6 [file gyae041_suppl_supplementary_datas_sd6.docx]

**Supplementary data (SD6): Assessing responses to heat in a range shifting nocturnal arboreal small mammal**

Hensley et al. 2023


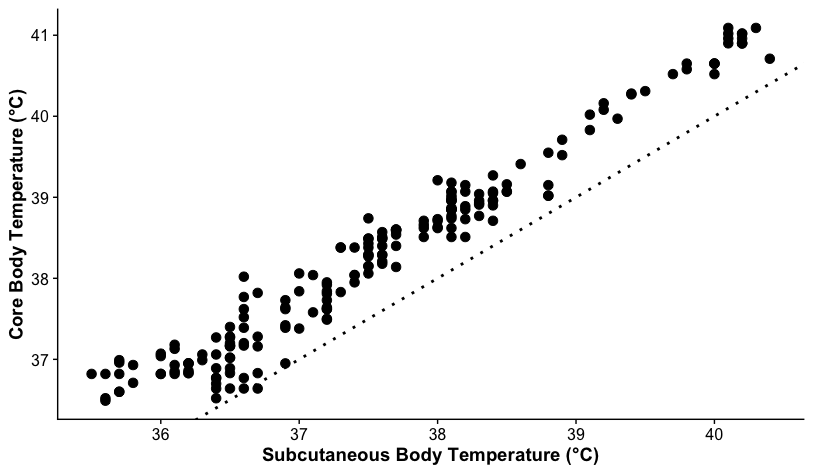


Core body temperature (measured via implanted temperature sensitive data loggers) as a function of subcutaneous body temperature (obtained from PIT-tags injected interscapularly) in southern flying squirrels (*Glaucomys volans*, N=3) measured during flow-through respirometry experiments. The dotted line indicates equality between the two values. Subcutaneous temperatures consistently underestimated core body temperature (mean -0.72°C±0.25).
